# Effects of repetitive transcranial magnetic stimulation on inhibitory control in first-episode schizophrenia: behavioral and neural mechanisms

**DOI:** 10.3389/fpsyt.2024.1496562

**Published:** 2024-11-04

**Authors:** Sihang Yu, Shuai Wang, Hang Sun

**Affiliations:** ^1^ School of Computer Science and Engineering, Xi’an Technological University, Xi’an, China; ^2^ School of Life Science and Technology, Xi’an Jiaotong University, Xi’an, China; ^3^ Football school of Xi’an Physical Education University, Xi’an, China

**Keywords:** rTMS, schizophrenia, inhibitory control, cognitive deficits, DLPFC, fMRI, brain activation

## Abstract

**Background:**

Inhibitory control deficits are a core feature of cognitive impairment in schizophrenia, associated with abnormal activation of key brain networks. Repetitive transcranial magnetic stimulation (rTMS) targeting the dorsolateral prefrontal cortex (DLPFC) may help improve inhibitory control, but its specific effects in schizophrenia remain uncertain.

**Methods:**

This study involved 150 participants divided into Real-rTMS, Sham-rTMS, and healthy control groups. Inhibitory control was assessed using the dual-choice oddball task, and task-based functional magnetic resonance imaging (fMRI) was employed to examine neural activity. The Real-rTMS group received active stimulation over the DLPFC, and the Sham group received placebo stimulation.

**Results:**

The Real-rTMS group exhibited significant improvements in both reaction times and accuracy compared to the Sham group, indicating enhanced inhibitory control. fMRI data showed that brain activity in regions such as the cerebellum, insula, thalamus, and prefrontal cortex was normalized in the Real-rTMS group, with activation patterns closely resembling those observed in healthy controls. Additionally, task-based fMRI revealed a restoration and further enhancement of negative activation in regions like the middle frontal gyrus and superior temporal gyrus, which helped reduce cognitive interference from irrelevant stimuli.

**Conclusion:**

rTMS targeting the DLPFC improves inhibitory control in schizophrenia by modulating both positive and negative brain activation patterns. These findings highlight the dual mechanism through which rTMS enhances cognitive control, offering a promising intervention for cognitive deficits in schizophrenia. Future research should explore the long-term effects of this modulation on broader cognitive functions.

## 1 Introduction

Schizophrenia (SCZ) is a chronic mental illness characterized by a wide range of cognitive, emotional, and behavioral disturbances (1, 2). Cognitive impairments, particularly deficits in inhibitory control, are among the most debilitating symptoms and significantly contribute to the functional impairments observed in patients (3, 4). Inhibitory control, the ability to suppress irrelevant or inappropriate responses, is crucial for goal-directed behavior, decision-making, and maintaining attention (5, 6). In schizophrenia, deficits in this area are associated with abnormal activity and connectivity within key brain networks, such as the default mode network (DMN) and the executive control network (7, 8). This dysfunction makes it difficult for individuals with schizophrenia to filter irrelevant information, resulting in cognitive overload, impaired judgment, and difficulties in completing daily tasks (8).

Addressing cognitive deficits, particularly inhibitory control, is essential for improving overall cognitive functioning and quality of life for patients with schizophrenia (9). Traditional pharmacological treatments, while effective in alleviating positive symptoms such as hallucinations and delusions, have shown limited efficacy in treating cognitive impairments (10, 11). This has led to increased interest in non-invasive neuromodulation techniques, such as repetitive transcranial magnetic stimulation (rTMS), as potential therapeutic interventions for cognitive deficits in schizophrenia (12).

rTMS is a non-invasive brain stimulation technique that uses magnetic fields to modulate neural activity in specific brain regions (13, 14). Over the past decade, rTMS has gained attention for its potential to enhance cognitive function in both healthy individuals and patients with neuropsychiatric disorders (14–16). In schizophrenia, rTMS has been explored primarily for its effects on negative symptoms and mood disorders, but growing evidence suggests it may also have a positive impact on cognitive deficits, including working memory, attention, and executive function (17, 18). However, despite its promise, the specific effects of rTMS on inhibitory control in schizophrenia remain underexplored, with mixed results reported across studies (19–21). Further research is needed to clarify whether rTMS can reliably improve inhibitory control in this population and to understand the neural mechanisms underlying such improvements.

The dorsolateral prefrontal cortex (DLPFC) has been identified as a critical region for cognitive control, including inhibitory control and working memory (22–24). rTMS targeting the DLPFC has been shown to modulate neural activity and improve cognitive performance in several clinical populations, including patients with depression, addiction, and neurodegenerative diseases (25–27). Given the role of the DLPFC in cognitive processing, it is hypothesized that rTMS applied to this region may help normalize brain function and enhance inhibitory control in schizophrenia (28). Studies involving other populations, such as those with substance use disorders, have demonstrated improvements in impulse control following rTMS, further supporting its potential application in schizophrenia (29, 30). Nevertheless, the precise neural mechanisms by which rTMS may influence inhibitory control in schizophrenia remain to be fully elucidated.

In this study, we aim to investigate the effects of rTMS on inhibitory control in patients with schizophrenia, focusing on both behavioral and neural mechanisms. We hypothesize that rTMS applied to the DLPFC will lead to significant improvements in inhibitory control, as measured by the dual-choice oddball task, a robust and widely used paradigm for assessing response inhibition (31). By comparing the performance of patients receiving real-rTMS with those receiving sham treatment, we aim to clarify whether rTMS can effectively enhance inhibitory control in this population. Additionally, we expect that these behavioral improvements in the Real group will be accompanied by changes in neural activity in regions associated with executive function, as measured by functional magnetic resonance imaging (fMRI). By comparing the Real and Sham groups, this study seeks to clarify the potential of rTMS as a targeted intervention for improving inhibitory control in schizophrenia and contribute to a deeper understanding of the neural mechanisms underlying these cognitive improvements.

## 2 Materials and methods

### 2.1 Participants

We enrolled 150 participants, with 50 in each group: Real-SCZ, Sham-SCZ, and healthy control (HC). All patients were at their first episode of schizophrenia and were diagnosed according to DSM-5 criteria (32). The two schizophrenia groups were matched for baseline clinical indicators and symptoms, with no significant differences between them. All patients were within two weeks of their initial exposure to antipsychotic treatment, with antipsychotic dosages converted to olanzapine equivalents using the Defined Daily Dose (DDD) system (33).

**Figure 1:**
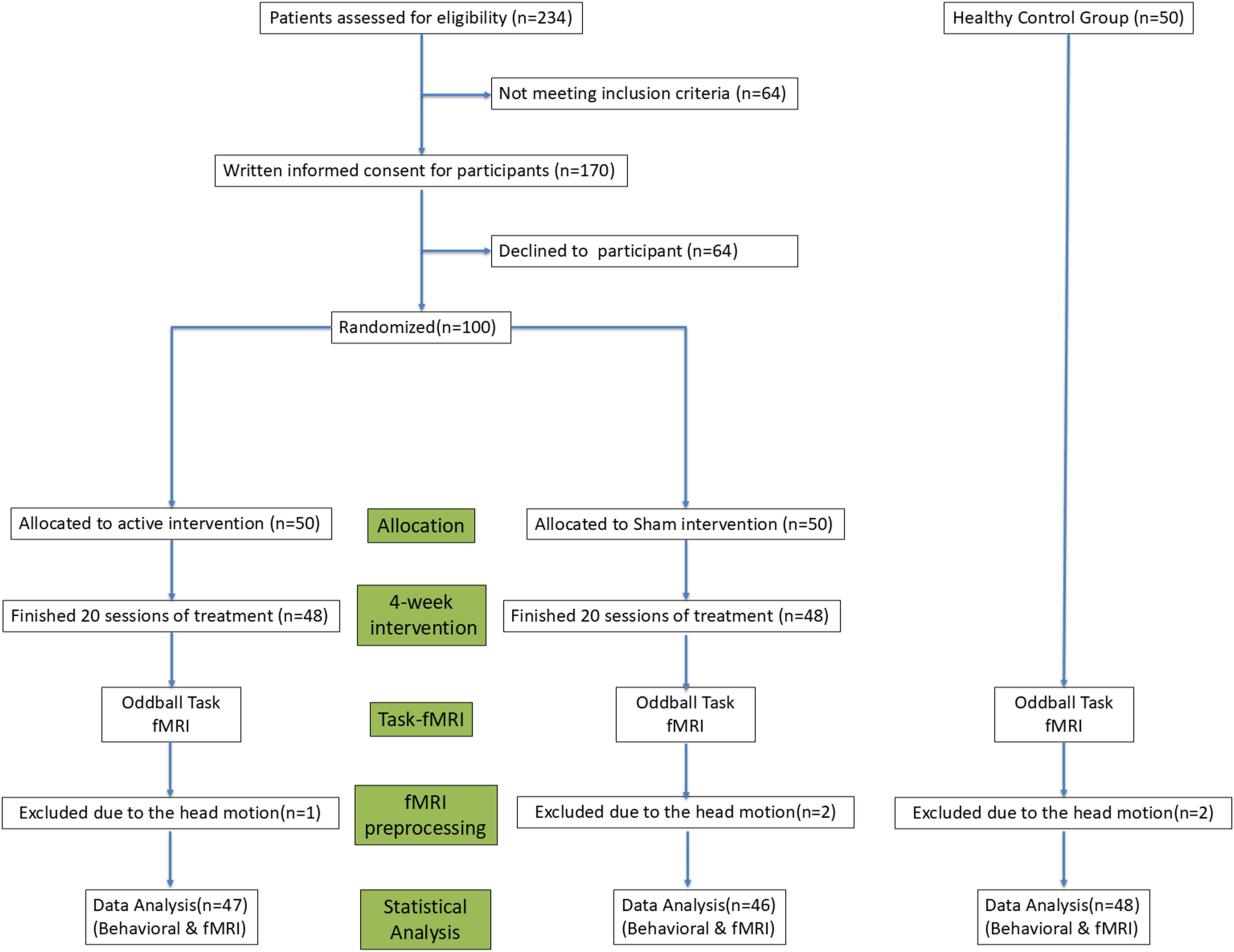
A flowchart outlining the experimental design of Experiment, detailing participant grouping, intervention protocols, and outcome measures.

The HC group was recruited through community advertisements and screened to exclude any history of psychiatric or neurological disorders. All participants were right-handed, had normal or corrected-to-normal vision, and were free from concurrent neurological or severe systemic illnesses. Exclusion criteria for all participants included a history of traumatic brain injury, substance use disorders, or contraindications to MRI. Both groups excluded individuals with an intelligence quotient (IQ) below 70, estimated by the Wechsler Abbreviated Scale of Intelligence (34). The study was approved by the local Research Ethics Committee and conducted in accordance with the Declaration of Helsinki. Written informed consent was obtained from all participants prior to the commencement of the study. The study was conducted from January 2023 to March 2024. This period included all phases of the study: participant recruitment, data collection, and the subsequent analysis of behavioral and neuroimaging data. The detailed experimental design can be seen in **Figure 1**.

### 2.2 Double-choice oddball task

The dual-choice oddball task requires participants to respond to two types of stimuli. One stimulus appears with a higher probability, approximately 85%, which is represented by the letter “W” in this experiment. The other stimulus appears with a lower probability, around 15%, and is represented by the letter “M” in this experiment. These stimuli are presented in random order, making the low-probability stimulus appear unexpectedly to the participant. The task requires participants to press the left key on the keyboard when they see the letter “W” and the right key when they see the letter “M.”

### 2.3 rTMS treatments

Participants received rTMS modulation over the left dorsolateral prefrontal cortex (DLPFC) according to the following protocol. Each rTMS session involved the delivery of 30 trains of TMS pulses at 10 Hz for 10 seconds (100 pulses per train) with a 20-second inter-train interval, resulting in a total of 3000 pulses per session for a total duration of 15 minutes. The stimulation intensity was set at 80% of the resting motor threshold, which was determined as the minimal intensity of stimulation that evoked an electromyographic response of 550 mV in the first dorsal interosseous muscle of the hand contralateral to the stimulated hemisphere in at least 5 out of 10 trials. For individualized stimulation (35), the MNI coordinates of the left DLPFC (approximately (–26, 38, 44), based on prior studies targeting this region) were adjusted for each participant using the inverse normalization method embedded in SPM12 (i.e., normalization based on the inverse deformation field). All rTMS sessions were guided by a neuronavigation system to ensure precise targeting. Once the location of the stimulation target was determined, the coordinates were entered into the neuronavigation system, which provided continuous guidance throughout the sessions, ensuring precise and consistent delivery of stimulation to the designated cortical area. For the Sham group, the rTMS coil was positioned at the same MNI coordinates of the left DLPFC as in the Real group, and the neuronavigation system was used to guide the coil placement. However, the coil was angled at 90 degrees away from the scalp, preventing direct brain stimulation while still producing auditory clicks and scalp sensations similar to the Real rTMS treatment. This setup ensured that participants in the Sham group experienced sensory feedback comparable to the Real group, such as the sound of the TMS pulses and the mild scalp sensations, without receiving active stimulation to the brain. This approach was used to maintain blinding of the participants and minimize placebo effects.

### 2.4 Task-based fMRI data acquisition and processing

Functional MRI data were collected during the oddball task using a Philips 3-Tesla MRI scanner with a 32-channel head coil. BOLD (Blood Oxygen Level Dependent) signals were acquired using an echo-planar imaging (EPI) sequence with the following parameters: repetition time (TR) = 2000 ms, echo time (TE) = 30 ms, flip angle = 90°, field of view (FOV) = 220 mm × 220 mm, slice thickness = 3 mm, and 45 axial slices covering the entire brain. Each fMRI session included both task and rest periods. Preprocessing of fMRI data was conducted using SPM12 (Statistical Parametric Mapping). The preprocessing steps included discarding the first 10 time points, slice timing correction, realignment, normalization to the MNI template, and spatial smoothing with a 6 mm full-width at half-maximum (FWHM) Gaussian kernel.

### 2.5 Task-based fMRI data analysis

The fMRI data were analyzed using a mixed-effects model in a two-stage process. In the first stage, the stimulus sequence corresponding to letter presentations was organized into distinct blocks and convolved with the hemodynamic response function (HRF). A general linear model (GLM) was then applied to identify brain regions showing significantly higher or lower activity in response to the presentation of the letter “M” compared to the control condition (“W”). To minimize the effects of head motion on the results, six motion parameters derived from the preprocessing stage were included in the GLM.

In the second stage, several steps were followed. First, for each experimental condition (HC, Real, and Sham), one-sample t-tests were performed to examine the overall brain activation patterns elicited by the dual-choice oddball task. Second, a one-way ANOVA was conducted to assess significant differences in brain activation patterns across the three experimental conditions. *Post-hoc* tests were performed to further explore pairwise comparisons between the conditions. Lastly, brain activation values were extracted from regions showing significant differences between the three groups, and detailed charts were generated to visually present these differences.

### 2.6 Behavioral data analysis

For the behavioral results of the dual-choice oddball task, including the accuracy difference between deviant and standard stimuli (deviant - standard) and the reaction time difference between deviant and standard stimuli (deviant - standard), a one-way ANOVA was conducted to compare the three groups (HC, Real, and Sham). The significance threshold was set at p < 0.05, with FDR correction applied for multiple comparisons. *Post-hoc* pairwise comparisons were corrected using the Bonferroni method, with the adjusted significance threshold set at 0.05.

For the neuroimaging data of the dual-choice oddball task, multiple comparison corrections were also applied using the FDR standard. For F-tests comparing activations across the three groups, as well as *post-hoc* pairwise comparisons, the statistical threshold was set at pFDR < 0.05.

## 3 Results

### 3.1 Demographic and clinical characteristics

Throughout the intervention phase, two participants in the Real group and two in the Sham group did not complete the treatment. Additionally, issues with data quality necessitated further exclusions: excessive head motion during fMRI scanning resulted in the exclusion of one participant from the Real group, two from the Sham group, and two from the Healthy Control group. Consequently, the analysis was conducted with a final sample of 141 participants, distributed as follows: 47 in the Real group, 46 in the Sham group, and 48 in the HC group. Comparisons between the three groups (Real, Sham, and HC) revealed no significant differences in age, gender, handedness, or frame-wise displacement. However, the HC group had significantly longer years of education compared to the schizophrenia (SZ) groups (p < 0.001). Furthermore, comparisons between the two SZ groups (Real and Sham) indicated no significant differences in duration of illness or in Positive and Negative Syndrome Scale (PANSS) scores, including the Positive score, Negative score, General score, and the Total score. Detailed demographic and clinical information for each group can be found in **Table 1**.

**Table 1:** Demographic and clinical characteristics of the HC and SZ.

Characteristic	HC (48)	SZ-Real (47)	SZ-Real (47)	*P* value	*P* value
Age: years	23.8±4.1	24.3±4.7	24.2±4.4	0.10 ^a^	0.67 ^b^
Gender (male/female)	24/24	23/24	23/23	1 ^a^	1
Education: years	15.5±1.9	12.5±2.8	12.3±2.3	<0.001 ^a^	0.91 ^b^
Handedness (right/left)	48/0	47/0	46/0	NA	NA
Frame-wise displacement	0.26±0.33	0.28±0.41	0.28±0.46	0.23 ^a^	0.94 ^b^
Duration of illness: month	NA	2.9±2.1	2.8±2.2	NA	0.46 ^b^
PANSS scores					
Positive score	NA	22.3±7.1	22.4±7.2	NA	0.53 ^b^
Negative score	NA	22.7±6.2	22.8±7.2	NA	0.71 ^b^
General score	NA	47.5±8.2	47.3±8.1	NA	0.89 ^b^
Total score	NA	87.4±17.3	87.8±17.6	NA	0.81 ^b^
Oddball RT					
Standard(W)	462.72 ± 31.81	468.03 ± 27.68	536.48 ± 32.86	<0.001^c^	<0.001 ^b^
Deviant(M)	504.82 ± 29.65	537.62 ± 27.84	643.57 ± 34.33	<0.001 ^c^	<0.001 ^b^
Differences(M-W)	42.10 ± 39.41	69.59 ± 38.43	107.09 ± 44.73	<0.001 ^c^	<0.001 ^b^
Oddball Accuracy					
Standard(W)	91.7% ± 4.6%	85.9% ± 5.2%	86.0% ± 3.6%	<0.001 ^c^	<0.001 ^b^
Deviant(M)	89.5% ± 4.4%	77.2% ± 4.6%	71.4% ± 4.2%	<0.001 ^c^	<0.001 ^b^
Differences(M-W)	-0.022 ± 0.059	-0.087 ± 0.070	-0.146 ± 0.054	<0.001 ^c^	<0.001 ^b^

SZ, schizophrenia; HC, healthy controls; NA, not applicable; PANSS, the Positive and Negative Syndrome Scale.

^a^Value from the independent-samples t test or chi-square test between HC and patients.

^b^Value from the independent-samples t test between patients receiving Real and Sham rTMS.

^c^Value from the one-way ANOVA.

### 3.2 Dual-choice oddball behavioral performance

**Figure 2:**
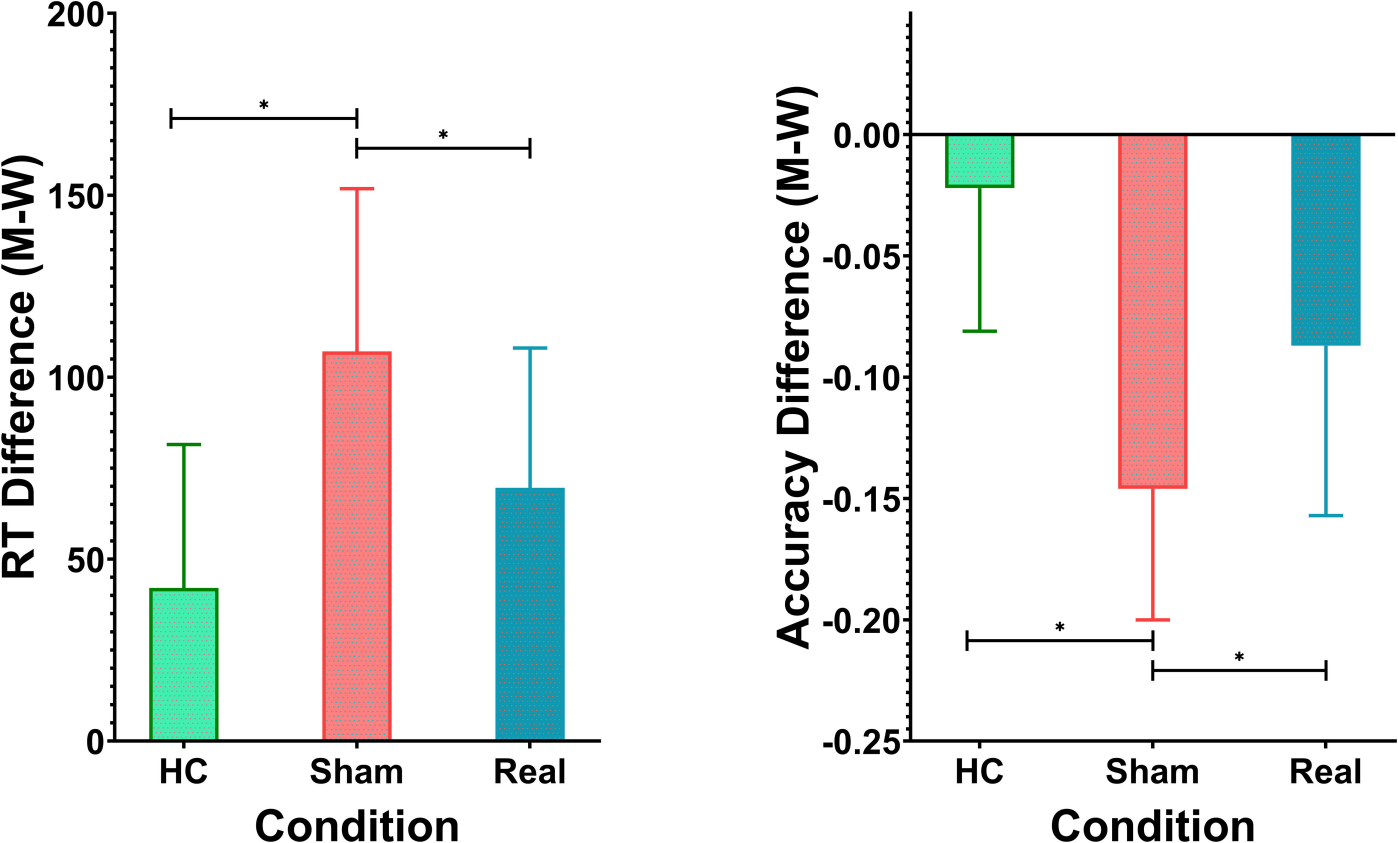
Behavioral Performance on the Dual-Choice Oddball Task. *p<0.01.

Participants’ performance was assessed using the dual-choice oddball task, measuring both reaction times (RT) and accuracy for standard and deviant stimuli. The healthy control (HC) group had shorter RTs for both standard stimuli (462.72 ± 31.81 ms) and deviant stimuli (504.82 ± 29.65 ms), with the smallest RT difference of 42.10 ± 39.41 ms. In comparison, the schizophrenia group receiving sham rTMS treatment exhibited the longest RTs for both standard stimuli (536.48 ± 32.86 ms) and deviant stimuli (643.57 ± 34.33 ms), resulting in the largest RT difference of 107.09 ± 44.73 ms. The Real rTMS group displayed intermediate RTs for standard stimuli (468.03 ± 27.68 ms) and deviant stimuli (537.62 ± 27.84 ms), with an RT difference of 69.59 ± 38.43 ms. A one-way ANOVA revealed significant variation in RT differences across the three groups (F_(2, 138)_ = 29.55, p < 0.0001). *Post-hoc* Tukey’s HSD tests further confirmed significant differences between all groups. The Sham group differed significantly from both the HC group (mean difference = 37.50 ms, p < 0.001) and the Real rTMS group (mean difference = -64.99 ms, p < 0.001). Additionally, the Real rTMS group showed a significant difference compared to the HC group (mean difference = -27.49 ms, p = 0.004). Results are shown in **Table 1**, **Figure 2**.

In terms of accuracy, the HC group performed with an average accuracy of 91.7% ± 4.6% for standard stimuli and 89.5% ± 4.4% for deviant stimuli. The Sham group displayed lower accuracy (86.0% ± 3.6% for standard and 71.4% ± 4.2% for deviant), while the Real group achieved 85.9% ± 5.2% accuracy for standard and 77.2% ± 4.6% for deviant stimuli. The accuracy difference (M - W) followed a similar pattern, with the Sham group showing the largest discrepancy (-0.146 ± 0.054), followed by the Real group (-0.087 ± 0.070) and the HC group (-0.022 ± 0.059). There was a highly significant effect of group on the accuracy difference (M - W), as indicated by ANOVA (F_(2, 138)_ = 48.96, p < 0.0001). *Post-hoc* Tukey’s tests revealed significant differences between all three groups. The Sham group had a significantly larger accuracy discrepancy compared to both the HC (mean difference = -0.1247, p < 0.001) and Real groups (mean difference = 0.0599, p < 0.001). The Real group also differed significantly from the HC group (mean difference = -0.0648, p < 0.001). Results are shown in **Table 1**, **Figure 1**.

### 3.3 Task-based fMRI activation patterns

**Figure 3:**
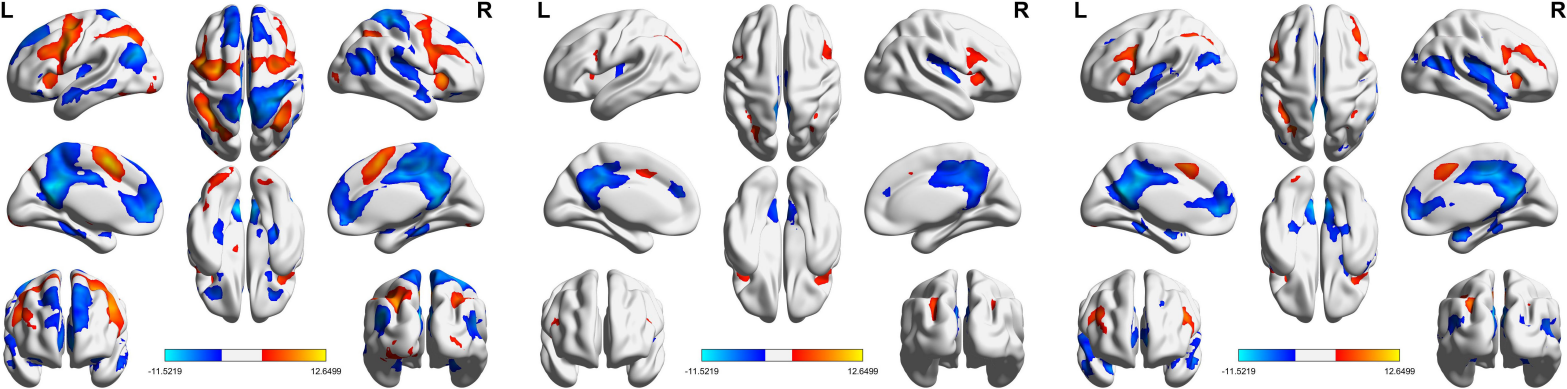
One-sample t-test results for each group.

**Figure 3** presents the results of the one-sample t-test, highlighting brain activation patterns in response to deviant stimuli compared to standard stimuli. The activation patterns were largely consistent across all groups, with key regions including the bilateral middle frontal gyrus, bilateral supplementary motor area, bilateral inferior frontal gyrus, left cingulate gyrus, right insula, and bilateral inferior parietal lobule.

**Figure 4:**
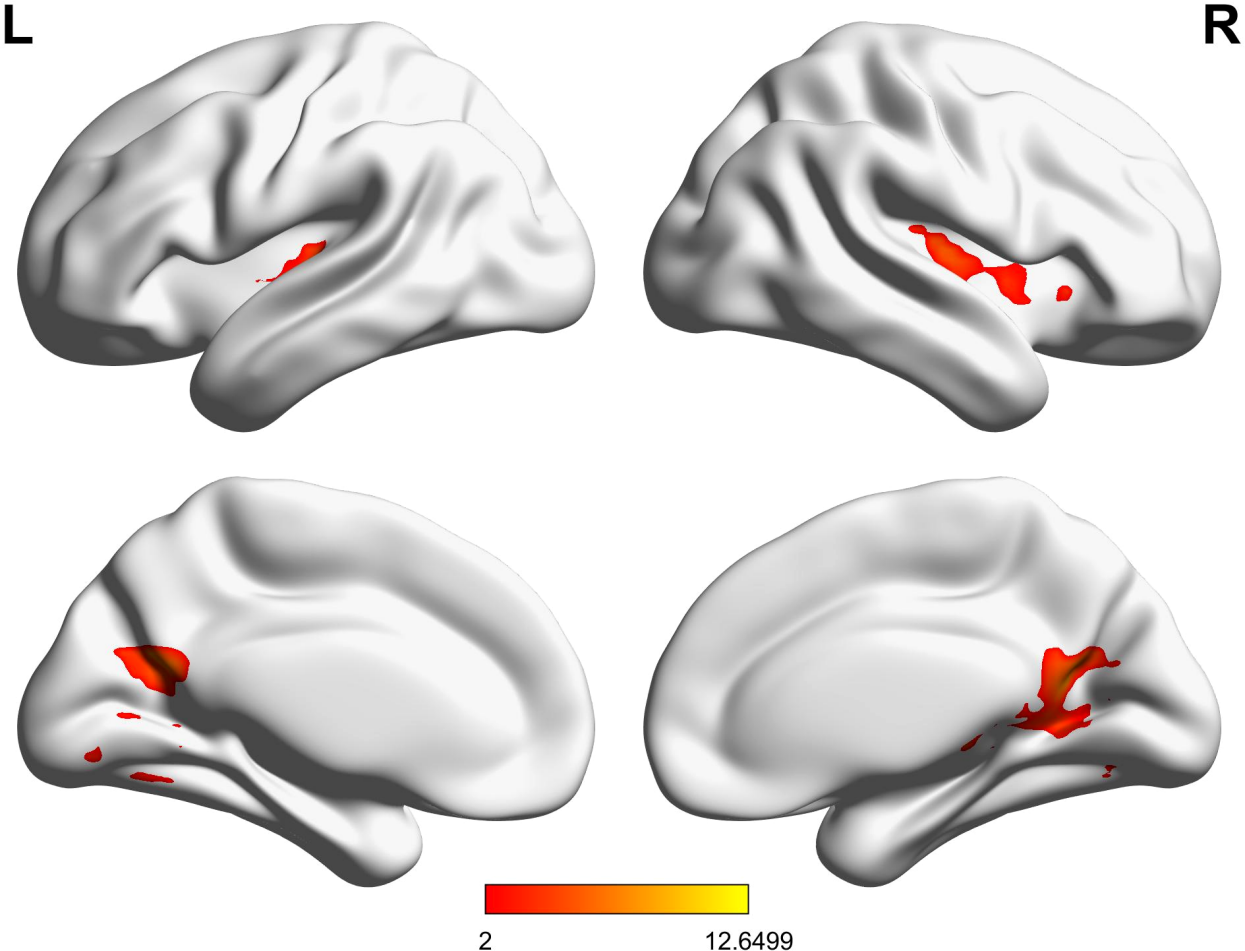
One-way ANOVA results comparing the three groups.

**Figure 5:**
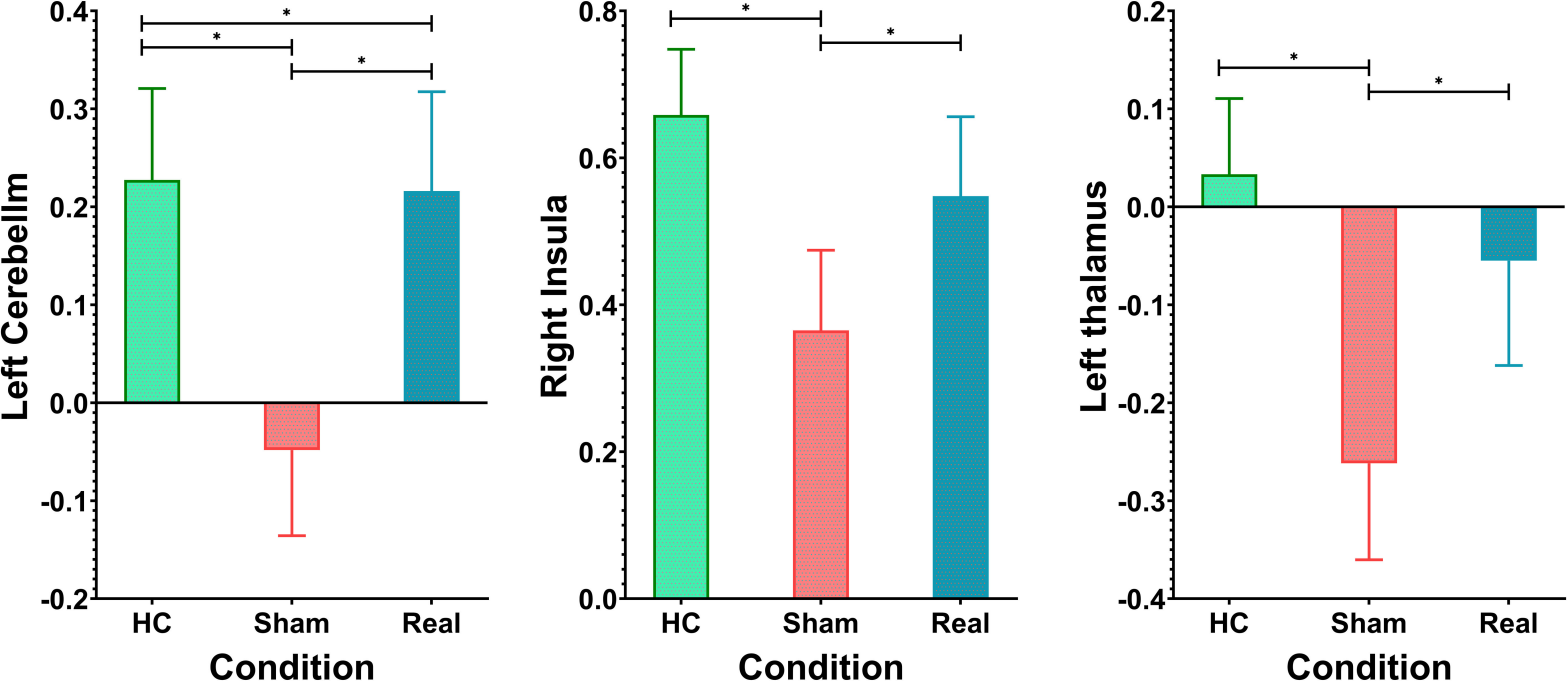
*Post-hoc* analysis of fMRI activations across the three groups. *p<0.01.

**Figure 4**, **Table 2** show the ANOVA results, revealing significant differences in brain responses among the three groups. The regions displaying these differences included the cerebellum, right insula, left thalamus, precuneus, superior temporal gyrus, and middle temporal gyrus. *Post-hoc* analyses, as illustrated in **Figure 5**, further identified specific group differences in the cerebellum, insula, and thalamus. The Sham group showed significantly reduced activity in these regions, while the Real group demonstrated a recovery in activation levels, approaching those of the healthy control group.

**Table 2:** Significant differences in brain responses among the three groups.

Regions-(ANOVA)	Number of voxels	Peak Coordinates (MNI)			*F-value*
		x	y	z	
Cerebellum					
Left	100	0	-51	-18	6.95
Insula					
Right	110	39	-15	9	6.64
Thalamus					
Left	103	-33	-24	6	6.97
Precuneus					
Right	579	15	-54	15	9.41
Superior Temporal Gyrus					
Right	53	48	-48	6	5.65
Middle Temporal Gyrus					
Left	136	-15	-33	24	7.64

Significance was set at P< 0.05 (false discovery rate corrected).

**Figure 6:**
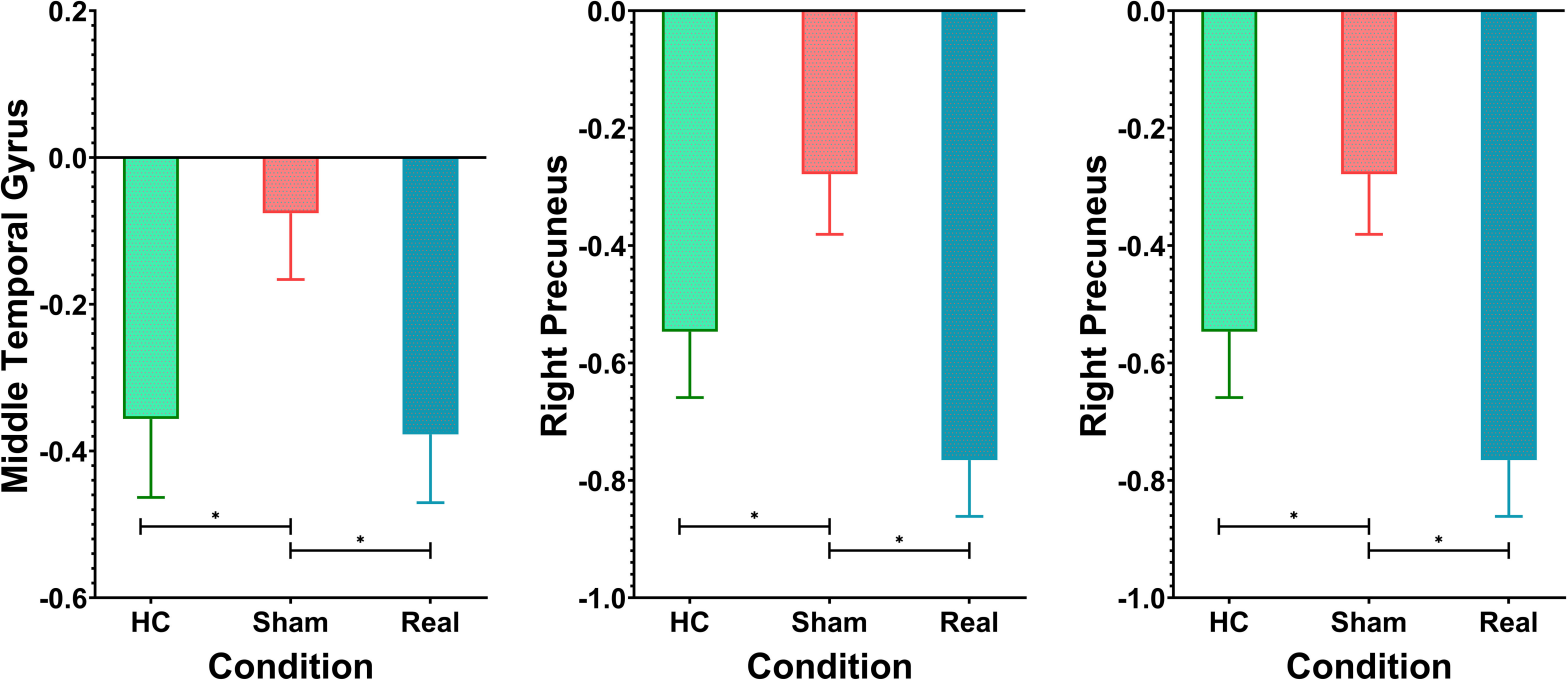
Additional patterns of fMRI activations across the three groups. *p<0.01.

A different pattern emerged in the middle temporal gyrus, precuneus, and superior temporal gyrus, as shown in **Figure 6**. These areas typically exhibit negative activation during the task under normal conditions. In the Sham group, this negative activation was reduced. However, after real rTMS treatment, negative activation in these regions was restored and even surpassed that of the healthy controls, reaching its peak level.

### 3.4 Correlation analysis

**Figure 7:**
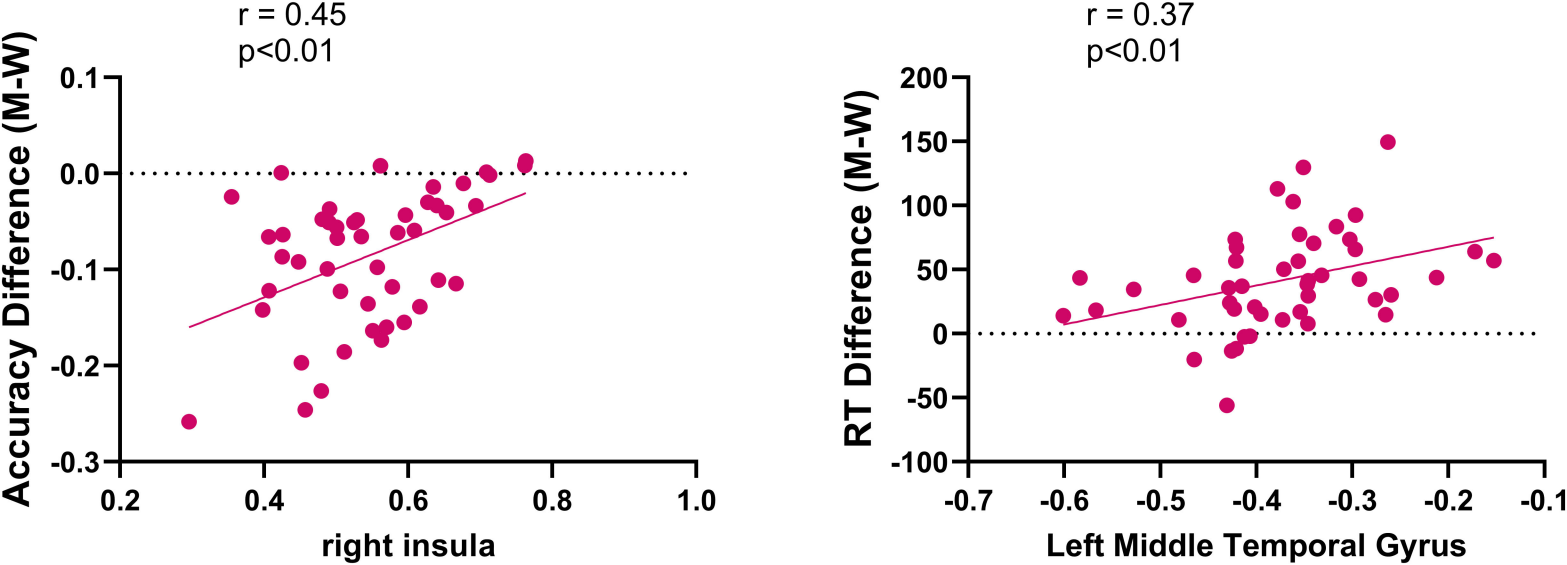
Correlations between fMRI activations and improvements in accuracy and reaction time in patients receiving real rTMS.

A significant positive correlation was observed between increased activation in the right insula and improvements in accuracy in the Real-rTMS group (r = 0.45, p < 0.01), besides, a positive correlation was also found between the increased de-activation within the middle temporal gyrus and the improvements of reaction time in the Real-rTMS group (r = 0.37, p < 0.01, **Figure 7**).

## 4 Discussion

The findings of this study provide compelling evidence that repetitive transcranial magnetic stimulation (rTMS) targeting the dorsolateral prefrontal cortex (DLPFC) can significantly improve inhibitory control in individuals with schizophrenia. The dual-choice oddball task revealed marked improvements in both reaction times and accuracy in the Real-rTMS group compared to the Sham group, supporting our hypothesis that rTMS can modulate inhibitory control processes. These behavioral improvements were further substantiated by functional magnetic resonance imaging (fMRI) results, which showed normalized brain activation in regions associated with cognitive control, such as the cerebellum, insula, thalamus, and prefrontal cortex. Together, these findings suggest that rTMS may act as a promising non-invasive intervention for cognitive deficits in schizophrenia, particularly for addressing inhibitory control impairments.

One of the key contributions of this study is the demonstration of rTMS’s dual mechanism of action in modulating both positive and negative brain activation patterns. The restoration of positive activation in areas such as the insula and thalamus, along with enhanced negative activation in regions like the middle temporal gyrus and precuneus, provides novel insights into how rTMS influences both excitatory and inhibitory processes in the brain. These results align with previous research suggesting that rTMS may help normalize the balance between excitatory and inhibitory neural activity (36–38). This balance is crucial for effective cognitive processing, and its restoration through rTMS could explain the observed improvements in task performance.

From a clinical perspective, the improvements in inhibitory control observed in the Real-rTMS group are particularly significant for early-stage schizophrenia. Cognitive impairments in schizophrenia, especially deficits in inhibitory control, tend to emerge early in the course of the illness and worsen over time (39, 40). Early interventions that target these impairments could have a substantial impact on long-term outcomes, helping to preserve cognitive function and improve overall prognosis (40, 41). Our findings suggest that rTMS could be a valuable tool for early cognitive interventions in schizophrenia, particularly for patients in the first episode of psychosis. Future research should explore the long-term effects of rTMS on cognitive outcomes in schizophrenia, as well as its potential to prevent the progression of cognitive decline.

The neurobiological underpinnings of these cognitive improvements provide further support for the use of rTMS in schizophrenia. The disconnection hypothesis of schizophrenia, which posits that cognitive impairments arise from disrupted connectivity between brain regions, is supported by our fMRI findings (42, 43). Specifically, the Real-rTMS group demonstrated more normalized functional connectivity patterns in regions associated with executive function and inhibitory control, suggesting that rTMS may enhance neuroplasticity and restore functional brain networks. This neuroplastic effect of rTMS could be especially beneficial in early-stage schizophrenia, where cognitive networks are more malleable and responsive to intervention (44, 45).

While this study provides important insights into the effects of rTMS on inhibitory control in schizophrenia, several limitations should be acknowledged. One of the primary limitations of this study is the lack of a comprehensive cognitive battery, such as the Brief Assessment of Cognition in Schizophrenia (BACS) or the MATRICS Consensus Cognitive Battery (MCCB) (46, 47). Without a standardized cognitive assessment, it remains unclear whether the Real and Sham groups differed in other cognitive domains beyond inhibitory control. For example, it is possible that the Sham group’s inferior performance could be due to broader cognitive impairments unrelated to the rTMS intervention. Future studies should incorporate a more comprehensive assessment of cognitive function to ensure that improvements are specific to inhibitory control rather than a general cognitive enhancement. Additionally, the short-term nature of the rTMS intervention limits the conclusions that can be drawn about the long-term efficacy of rTMS in improving cognitive function in schizophrenia. While the immediate post-treatment effects are promising, it is unclear whether these improvements are sustained over time. Longitudinal studies with follow-up assessments are necessary to determine whether rTMS provides lasting cognitive benefits and to explore the potential for maintenance or booster sessions to prolong the effects of the intervention. Another limitation relates to the sample size and demographic homogeneity of the participants. Although the sample size was sufficient for detecting significant effects, a larger and more diverse sample would allow for greater generalizability of the findings. In particular, future research should explore whether factors such as illness chronicity, medication status, and baseline cognitive function influence the effectiveness of rTMS. Stratifying participants based on these factors could provide a clearer understanding of who stands to benefit the most from rTMS and how the intervention can be tailored to individual patients.

The results of this study open several avenues for future research. First, given the neuroplastic effects of rTMS observed in this study, future investigations should explore the combination of rTMS with other therapeutic interventions that target cognitive deficits, such as cognitive remediation therapy (CRT) or pharmacological agents aimed at enhancing neuroplasticity. Combining these approaches could yield additive or synergistic effects, leading to greater improvements in cognitive function and functional outcomes. Second, this study focused on the DLPFC as the primary target for rTMS, but other brain regions involved in inhibitory control, such as the anterior cingulate cortex (ACC) or orbitofrontal cortex (OFC), may also be important targets for modulation. Future studies should explore whether stimulating these regions in combination with the DLPFC could lead to even greater improvements in cognitive function. Finally, research should also investigate the potential role of rTMS in other cognitive domains beyond inhibitory control, such as working memory, attention, and executive function. Understanding the broader cognitive effects of rTMS could help refine its use as a comprehensive cognitive intervention for schizophrenia and other neuropsychiatric conditions.

In summary, this study demonstrates that rTMS targeting the DLPFC can significantly improve inhibitory control in individuals with schizophrenia, with both behavioral and neural evidence supporting its efficacy. The normalization of brain activity in key regions associated with cognitive control suggests that rTMS may help restore functional connectivity and enhance neuroplasticity in patients with schizophrenia. While these findings are promising, further research is needed to address the limitations of the current study and to explore the long-term efficacy and broader cognitive effects of rTMS. Ultimately, this research contributes to the growing body of evidence supporting rTMS as a potential therapeutic tool for addressing the cognitive deficits that significantly impair the lives of individuals with schizophrenia.

## Data Availability

The raw data supporting the conclusions of this article will be made available by the authors, without undue reservation.
